# Environmental Stress and Plants 2.0

**DOI:** 10.3390/ijms241512413

**Published:** 2023-08-04

**Authors:** Luigi Parrotta, Lavinia Mareri, Giampiero Cai

**Affiliations:** 1 Department of Biological, Geological and Environmental Sciences, University of Bologna, Via Irnerio 42, 40126 Bologna, Italy; 2 Department of Life Sciences, University of Siena, Via P.A. Mattioli 4, 53100 Siena, Italy; lavinia.mareri@unisi.it (L.M.); cai@unisi.it (G.C.)

Following the success of our previous edition [1], this Special Issue ‘Environmental Stress and Plants 2.0’ includes 23 original articles and 3 reviews. The high level of participation and the large number of reports show that plant researchers are interested in this topic. We provide a brief overview of the papers, which include original articles (categorized by stress type) as well as reviews.

**Drought stress**

Global warming is causing decreased precipitation, and there is an urgent need to study the effects of water scarcity on natural and artificial ecosystems. The expected increase in global temperature in the twenty-first century, along with the disruption of soil water retention, will increase the possibility of frequent droughts and desertification of agriculture areas. As a result, the search for biochemical indicators of drought-resistant cultivars is crucial. Lahuta and colleagues [2] focused on *Pisum sativum* L. plant shoots subjected to repeated short-term soil drought and re-watering. Pea plant metabolite analysis showed alterations in amino acid and soluble carbohydrate concentrations. Proline, γ-aminobutyric acid (GABA), branched-chain amino acids, hydroxyproline, serine, myo-inositol, and raffinose increased during drought and subsequently dropped upon re-watering. Proline accumulation was a significant feature of pea plant drought memory. Drought additionally presents a danger to wheat output; hence, boosting wheat yield in drought conditions is a critical research aim. Lan et al. [3] investigated several lines of *Triticum aestivum* L., evaluating 13 traits until maturity. Early drought stress produced higher yields in the rye genotypes than in the other genotypes, while late drought produced no discernible patterns and early root vigour was an important trait in wheat breeding for drought tolerance. Physiological responses were investigated by Hernandez and Park [4] who investigated the effects of water deficit stress on leaf anatomical and physiological traits, as well as stem starch content, in *Quercus acutissima* Carruth and *Quercus serrata* Murray seedlings subjected to well-watered (WW) and water deficit stress (WS) treatments. Drought caused changes in trichrome density, trichrome-to-stomata ratio, mesophyll thickness, vein density, distance and loopiness, vessel diameter, transpiration (E), stomatal conductance (g_s_), water use efficiency (WUE), and starch content. These changes suggest an important role for *Q. acutissima* and *Q. serrata* seedling survival in water-deficient environments, though *Q. serrata* may be more resistant to prolonged water stress than *Q. acutissima*. A subsequent paper focused on genetic responses. Chakraborty and colleagues [5] investigated pearl millet (*Pennisetum glaucum* L.), an important crop for sustaining food and fodder production in arid and semi-arid ecosystems. The cellular and molecular basis of drought resistance was studied using functional genomics techniques to increase crop production. A total of 74 drought-responsive genes were studied and classified into five major phylogenetic groups and eight functional groups, which included ABA signalling, hormone signalling, ion and osmotic homeostasis, TF-mediated regulation, molecular adaptation, signal transduction, physiological adaptation, and detoxification. The functionally validated genes are promising candidates for backcross breeding, genomic selection, and gene editing programs in pearl millet and other millet crops to increase yield in drought-prone arid and semi-arid ecosystems.

**Temperature stress**

Temperature fluctuations are a prominent feature of climate change. Several researchers have investigated genetic and molecular aspects of plant response to temperature stress. OsFKBP20-1b, a plant-specific cyclophilin protein, regulates pre-mRNA splicing in rice (*Oryza sativa*) under stress conditions. Park et al. [6] demonstrated that OsFKBP20-1b is SUMOylated and interacts with OsSUMO1 and OsSUMO2 in the nucleus and cytoplasm. Under heat stress, the abundance of an OsFKBP20-1b-GFP fusion protein increased in nuclear speckles and cytoplasmic foci, whereas the heat responsiveness was reduced in the presence of the less SUMOylated 5KR OsFKBP20-1b-GFP mutant. Heat stress increased the accumulation of endogenous SUMOylated OsFKBP20-1b *in planta*, suggesting that SUMOylation regulates OsFKBP20-1b post-translationally and that the modification is critical for proper RNA processing in response to heat stress in rice. Cold stress is a crucial factor limiting temperate crop yield and geographical distribution, as it alters several biological and molecular mechanisms. Wang et al. [7] found that the wheat elongator subunit 4 (TaELP4) of wheat (*Triticum aestivum* L.) negatively regulates freezing tolerance via ethylene signalling. Elongator is a six-subunit complex that participates in plant development and defence responses by acting as a histone acetyltransferase. the *TaELP4* promoter contains cold response elements and is up-regulated by freezing stress, implying that *TaELP4* inhibited plant responses to freezing stress by increasing histone acetylation levels. A subsequent paper focuses on cold stress; specifically, *Juglans mandshurica*, which has high freezing resistance and can survive temperatures as low as −40 °C, was used as an important freeze-tolerant germplasm resource. In the study of Zhao et al. [8], the *J. mandshurica* genome was analysed to identify 184 *AP2/ERF* genes (APETALA2/ethylene-responsive factors regulating plant development, growth, and response to biotic/abiotic stress), which were classified into five subfamilies (*JmAP2*, *JmRAV*, *JmSoloist*, *JmDREB*, and *JmERF*). This study sheds new light on the role of the *JmAP2/ERF* gene in cold stress response, paving the way for functional validation of *JmAP2/ERF* transcription factors and their application in the genetic improvement of *Juglans* and other tree species. Cold resistance is the focus of the paper of Zhao et al. [9]. The C-Repeat Binding Factor (*CBF*) gene family has been identified and characterized in multiple plant species, and it plays an important role in responding to low temperatures. The *CBF* gene family was analysed in *Acer truncatum*, *Acer pseudosieboldianum*, and *Acer yangbiense*. The findings revealed that sixteen CBF genes (five *ApseCBFs*, four *AcyanCBFs*, and seven *AtruCBFs*) were unevenly distributed across the chromosomes, with the majority of *CBF* genes mapped on chromosomes 2 and 11. The comprehensive analysis will improve our understanding of the potential functions of the *CBF* genes in cold resistance.

**Salt stress**

Excess salinity is also an environmental threat to crops because it alters plant performance by causing metabolic damage, ion toxicity, secondary oxidative stress, and osmotic stress. Stefanov et al. [10] investigated the functions of the photosynthetic apparatus of C3 (*Pisum sativum* L.) and C4 (*Zea mays* L.) plants after treatment with different NaCl concentrations (0–200 mM). The data showed that C4 plants had lower photosynthetic structure density (RC/CSo), a larger relative size of the plastoquinone (PQ) pool (N), and higher electron transport capacity and photosynthetic rate (parameter R_Fd_) than C3 plants. The data also revealed that NaCl treatment reduced the density of photosynthetic structures and the relative size of the PQ pool, as well as electron transport to the PSI end electron acceptors, with the effects being stronger in pea than maize. *Hemerocallis fulva* is a perennial herbaceous plant with fleshy roots, a wide distribution, and high adaptability. Salt stress is a serious abiotic stresses limiting *H. fulva* growth and production. In the study of Zhou et al. [11], miRNA expression was significantly different in the roots and leaves of *H. fulva*. miRNAs are non-coding small RNAs that play a post-transcriptional regulatory role in plant growth development and response to abiotic stress. The findings suggest that non-coding small RNAs and their target genes involved in the phytohormone signalling, Ca^2+^ signalling, and oxidative defence signalling pathways are involved in the response of *H. fulva* to NaCl stress.

**UV and high light stresses**

UV radiation, particularly UV-B, has long been thought to be a plant stressor, causing DNA, protein, and membrane damage. Oat is a worldwide food and forage crop. It is also an important forage grass in China’s plateau regions, where ultraviolet radiation and sunlight are abundant. Yin et al. [12] analysed eight reference genes (sulfite oxidase, SUOX; victorin binding protein, VBP; actin-encoding, Actin1; protein PSK SIMULATOR 1-like, PSKS1; TATA-binding protein 2-like, TBP2; ubiquitin-conjugating enzyme E2, UBC2; elongation factor 1-alpha, EF1-a; glyceraldehyde-3-phosphate dehydrogenase 1, GAPDH1). Under UV-B stress, the most stably expressed reference genes in the roots, stems, and leaves of oat were *EF1-a*, *TBP2*, and *PSKS1*, respectively; under high light stress, the most stably expressed reference genes were *PSKS1*, *UBC2*, and *PSKS1*, respectively. *PSKS1* was the most consistently expressed reference gene across all samples. This study emphasizes reference genes for accurate quantitative analysis of gene expression in various oat tissues under UV-B and high light stresses.

**Deficit of nutrients stress**

Soil nutrient limitation reduces plant growth, productivity, and quality and it is critical to make an early and specific diagnosis of a macronutrient deficiency. Courbet et al. [13] investigated the feasibility of using transcriptomic and metabolomic analysis of *Brassica napus* roots to characterize the effects of six individual macronutrient deprivations (N, Mg, P, S, K, and Ca). The findings revealed that all macronutrient deprivations resulted in a large modulation of the transcriptome and metabolome involved in various metabolic pathways, some of which were shared by all macronutrient deprivations.

**Herbicide stress**

Pesticide use is increasing, raising public concern about their negative effects on soil–plant–water systems and other environmental impacts. *Eleusine indica* (goosegrass) is a weed with a multi-herbicide tolerance/resistance biotype. Luo et al. [14] report a successful *Agrobacterium*-mediated transformation method for goosegrass, which allowed paraquat-resistant EiKCS gene overexpression in paraquat-susceptible goosegrass and stable inheritance of paraquat resistance in transgenic goosegrass lines. This method shed light on a potential mechanism for the evolution of paraquat-resistant goosegrass as well as a promising gene for manipulating paraquat-resistant plants; it will also be useful in future herbicide resistance research.

**Application of multiple stresses**

Multiple or combined stresses represent an unfavourable environmental condition that reduces crop yield. Rice is the world’s most important staple food, whose production is constrained by unfavourable environmental conditions. Habibpourmehraban et al. [15] investigated the effect of abscisic acid (ABA) pre-treatment on the tolerance of rice genotype after a 4-day exposure to combined drought, salt, and extreme temperature treatments. A total of 3285 proteins were identified and quantified among the four treatment groups (control, stressed, with/without ABA treatment). When compared to the control, pre-treatment with the ABA hormone significantly reduced leaf damage from combined abiotic stress at the proteome level. These findings suggest that exogenous ABA may have a priming effect on rice seedling tolerance to combined abiotic stress by influencing stress-responsive mechanisms in plants that rely on ABA signalling pathways. Because of unpredictable and intense rainfall, the frequency of waterlogging episodes has increased. Waterlogging memory is poorly known, as is its interaction with other climate change events such as elevated CO_2_ concentrations (e[CO_2_]). Zhou et al. [16] investigated the effects of e[CO_2_] and two rounds of waterlogging stress on the growth of cultivated (*Solanum lycopersicum*) and wild tomato (*S. pimpinellifolium*). The two rounds of treatments appeared to induce different acclimation strategies in the tomato genotypes. As demonstrated by decreased photosynthesis and biomass loss, *S. pimpinellifolium* responded more negatively to the first waterlogging than *S. lycopersicum*. Nonetheless, when waterlogging stress recurred, the two genotypes responded similarly, indicating that they could maintain a higher photosynthesis level compared to a single stress, particularly for the wild genotype. This demonstrated that waterlogging priming improved stress memory in both tomato genotypes. After a herbivore attack, white spruce (*Picea glauca*) emits monoterpenes that act as defensive signals and weapons. Perreca [17] and co-workers assessed the effects of drought and methyl jasmonate (MeJA) treatment. Compared to the control, MeJA treatment significantly increased monoterpene emission, but drought suppressed MeJA-induced emission. Stress altered the composition of the emission mixture, with drought increasing the proportion of oxygenated compounds and MeJA increasing the proportion of induced compounds such as linalool and (E)-β-ocimene. These findings show that monoterpene emission is sensitive to abiotic stress regimes. Fern–environment interactions have been extensively studied. Pietrak et al. [18] investigated the effects of salinity and full sunlight, as well as the combination of both stresses, on the growth and metabolic parameters of two hardy ferns (*Athyrium nipponicum* cv. Red Beauty and *Dryopteris erythrosora*). Hardy ferns are captivating ornamental plants that could provide antioxidants for the pharmaceutical, cosmetic, and food industries. The results showed that *D. erythrosora* has a higher adaptive potential to stresses than *A. nipponicum* cv. Red Beauty. These findings shed light on the physiological mechanisms underpinning hardy ferns’ sensitivity/tolerance to salinity, full sunlight, and combined stresses. Fruit shape and size are complex traits influenced by genetics and environmental factors. Zhang et al. [19] explored the mechanism of fruit shape and size development in cucumber. The authors measured the length and diameter of fruits, the length and diameter of cells, and the expression of related genes. In both lines, cell length and diameter were positively correlated with fruit length and diameter. These findings will aid in precision management by monitoring fruit growth and forecasting harvesting times under varying temperature and light conditions. Liquorice (*Glycyrrhiza inflata*) is a plant in the genus Leguminosa Glycyrrhiza linn that has a variety of commercial uses in the pharmaceutical, cosmetics, and food industries. *Glycyrrhiza inflata* Bat. demonstrated strong abiotic stress resistance, with terpenoids and flavonoids being the most important bioactive components. Yang and co-workers [20] identified 2 SnRK1 (Sucrose non-fermenting-1-related protein kinase-1)-catalytic-subunit-encoding genes (GiSnRK1α1 and GiSnRK1α2) and 21 FLZ (FCS-like zinc finger proteins) genes involved in various processes of plant growth and stress responses. *GiFLZs* are actively responsive to methyl jasmonic acid (MeJA) and abscisic acid (ABA) treatments, and several *GiFLZs* and *GiSnRK1α1* are regulated by drought and saline–alkaline stresses. These data provide information on the SnRK1 catalytic subunit and the FLZ proteins in liquorice for future functional characterization. Du et al. [21] carried out a genome-wide analysis of wheat (*Triticum aestivum* L.) GATA transcription factor genes to uncover their molecular evolutionary characteristics and roles in salt and drought tolerance. Plant growth, development, morphogenesis, and stress response are all influenced by GATA transcription factor genes. There are 79 *TaGATA* genes with a conserved GATA domain that have been identified and classified into four subfamilies. *TaGATA* genes were highly expressed in leaves and in response to drought and salt stress, according to transcriptional expression analysis. *TaGATAs* could improve drought and salt tolerance through direct interaction of the DNA-binding motif of GATA transcription factors with the photomorphogenesis-related protein TaCOP9-5A. These findings laid the groundwork for future research into the molecular evolution and functional characterization of the plant GATA gene family in response to abiotic stresses. The JAZ protein and MYC transcription factors are essential for plant response to external environmental changes, growth, and development because they are key regulators of the jasmonate (JA) signal transduction pathway. Wang et al. [22] identified 18 *StJAZs* and 12 *StMYCs* in potatoes. The *StJAZ* genes’ chromosomal position, phylogenetic development, gene structure, and promoter cis-acting parts were studied. The expression patterns of *StMYC6*, *StJAZ11*, and *StJAZ16* were different under mannitol-induced drought or salinity treatment, indicating that the JAZ protein and MYC transcription factor may be involved in the response of potatoes to abiotic stress. This opens a new prospect for potato genetic improvement in response to environmental stress. Using the barley pangenome and the Tibetan Lasa Goumang genome, An et al. [23] determined the number of members, genetic similarity, and phylogeny of histone methyltransferase (HMT) and histone demethylase (HDM) families in barley. The findings revealed that HMT and HDM were highly conserved during barley domestication, but there were some differences in the Lasa Goumang SDG subfamily. *HvHMTs* and *HvHDMs* were highly expressed in specific tissues and showed complex expression patterns in response to various stress treatments. In barley, HMT and HDM are highly conserved and play an important role in growth and development during abiotic stresses. These findings shed light on the origins and evolutionary history of plant *HvHMTs* and *HvHDMs*, laying the groundwork for future research in barley. *Mikania micrantha* is a rapidly spreading tropical vine that has infiltrated South China’s coastal areas, causing significant economic and environmental damage. *M. micrantha* has rapid stem growth, which may be due to its higher number of genes involved in auxin signalling and transport pathways, as well as its ability to synthesize more auxin under adverse conditions to promote or maintain stem growth. The PIN-FORMED (PIN) auxin efflux carrier gene family regulates the growth of various plant tissues by regulating the polar transport of auxin. Chen et al. [24] highlighted the structural features and stress response patterns of the PIN gene family, as well as providing insights for future research into the molecular mechanism of auxin-regulated growth in *M. micrantha*.

**Reviews**

Progesterone is a steroid hormone that supports important functions in mammals, and research into its physiological functions in plants has grown in recent years. Li et al. [25] reviewed the regulatory functions of progesterone in plant growth and development, as well as its response to stress. Progesterone is abundant in plants and can regulate a variety of physiological processes, even at low concentrations. Because progesterone has similar properties to plant hormones, it is expected to become a plant hormone candidate. Regrettably, most current research on progesterone in plants is at the physiological level, and more molecular research is required to clarify progesterone signalling pathways. Rice is one of the major food crops worldwide and heat stress has a significant impact on its yield and quality. Clarifying the molecular mechanism of heat tolerance in rice varieties is therefore critical. Liu et al. [26] provide new insights for future research. Drought stress has a negative impact on plants’ physiological, genetic, biochemical, and morphological characteristics. Drought also affects flower development and pollen sterility, resulting in lower seed production and fruit quality, in particular in tomato (*Solanum lycopersicum* L.) plants. Conti and co-workers [27] summarized the contribution of specific physio-molecular traits to drought tolerance and how they differ. The core of this review is a critical assessment of the importance of conserving and valorising tomato biodiversity as a gene pool against abiotic stress conditions. The review covers the gene expression, biochemistry, metabolism, and physiology of tomato cultivars, emphasizing the importance of tomato biodiversity for an efficient drought response and preservation of fruit quality levels.

Finally, abiotic stresses will continue to be an ongoing concern for the natural environment and agriculture. Plant and crop productivity is affected by a variety of unfavourable conditions, limiting food supply and increasing production costs. In the face of oncoming climate change, the current scarcity of arable land, the expanding global population, and diminishing water supplies make it difficult to produce more. Increasing plant biology knowledge and agricultural improvement are strategic goals for better understanding abiotic stress responses, identifying stress protection networks, and designing environmentally stable crops that are more productive and adaptable to environmental changes.

## References

[1] Mareri L., Parrotta L., Cai G. (2022). Environmental Stress and Plants. Int. J. Mol. Sci..

[2] Lahuta L.B., Szablinska-Piernik J., Horbowicz M. (2022). Changes in Metabolic Profiles of Pea (*Pisum sativum* L.) as a Result of Repeated Short-Term Soil Drought and Subsequent Re-Watering. Int. J. Mol. Sci..

[3] Lan Y., Chawade A., Kuktaite R., Johansson E. (2022). Climate Change Impact on Wheat Performance-Effects on Vigour, Plant Traits and Yield from Early and Late Drought Stress in Diverse Lines. Int. J. Mol. Sci..

[4] Hernandez J.O., Park B.B. (2022). The Leaf Trichome, Venation, and Mesophyll Structural Traits Play Important Roles in the Physiological Responses of Oak Seedlings to Water-Deficit Stress. Int. J. Mol. Sci..

[5] Chakraborty A., Viswanath A., Malipatil R., Semalaiyappan J., Shah P., Ronanki S., Rathore A., Singh S.P., Govindaraj M., Tonapi V.A. (2022). Identification of Candidate Genes Regulating Drought Tolerance in Pearl Millet. Int. J. Mol. Sci..

[6] Park H.J., Jung H.M., Lee A., Jo S.H., Lee H.J., Kim H.S., Jung C.K., Min S.R., Cho H.S. (2021). SUMO Modification of OsFKBP20-1b Is Integral to Proper Pre-mRNA Splicing upon Heat Stress in Rice. Int. J. Mol. Sci..

[7] Wang K., Zhai M., Han R., Wang X., Xu W., Zeng X., Qi G., Komatsuda T., Liu C. (2022). Wheat Elongator Subunit 4 Negatively Regulates Freezing Tolerance by Regulating Ethylene Accumulation. Int. J. Mol. Sci..

[8] Zhao M., Li Y., Zhang X., You X., Yu H., Guo R., Zhao X. (2022). Genome-Wide Identification of AP2/ERF Superfamily Genes in *Juglans mandshurica* and Expression Analysis under Cold Stress. Int. J. Mol. Sci..

[9] Zhao Q., Han R., Cai K., Yan H., Li Y., Qu G., Liu L., Zhao X. (2023). Identification and Analysis of the CBF Gene Family in Three Species of Acer under Cold Stress. Int. J. Mol. Sci..

[10] Stefanov M.A., Rashkov G.D., Apostolova E.L. (2022). Assessment of the Photosynthetic Apparatus Functions by Chlorophyll Fluorescence and P(700) Absorbance in C3 and C4 Plants under Physiological Conditions and under Salt Stress. Int. J. Mol. Sci..

[11] Zhou B., Gao X., Zhao F. (2023). Integration of mRNA and miRNA Analysis Reveals the Post-Transcriptional Regulation of Salt Stress Response in *Hemerocallis fulva*. Int. J. Mol. Sci..

[12] Yin H., Yin D., Zhang M., Gao Z., Tuluhong M., Li X., Li J., Li B., Cui G. (2022). Validation of Appropriate Reference Genes for qRT-PCR Normalization in Oat (*Avena sativa* L.) under UV-B and High-Light Stresses. Int. J. Mol. Sci..

[13] Courbet G., D’Oria A., Maillard A., Jing L., Pluchon S., Arkoun M., Pateyron S., Paysant Le Roux C., Diquelou S., Ourry A. (2021). Comparative Omics Analysis of *Brassica napus* Roots Subjected to Six Individual Macronutrient Deprivations Reveals Deficiency-Specific Genes and Metabolomic Profiles. Int. J. Mol. Sci..

[14] Luo Q., Chen S., Nian H., Ma Q., Ding Y., Hao Q., Wei J., Patel J.D., McElroy J.S., Liu Y. (2023). Establishment of an Efficient *Agrobacterium*-Mediated Genetic Transformation System to Enhance the Tolerance of the Paraquat Stress in Engineering Goosegrass (*Eleusine indica* L.). Int. J. Mol. Sci..

[15] Habibpourmehraban F., Wu Y., Masoomi-Aladizgeh F., Amirkhani A., Atwell B.J., Haynes P.A. (2023). Pre-Treatment of Rice Plants with ABA Makes Them More Tolerant to Multiple Abiotic Stress. Int. J. Mol. Sci..

[16] Zhou R., Jiang F., Yu X., Abdelhakim L., Li X., Rosenqvist E., Ottosen C.O., Wu Z. (2022). Dominant and Priming Role of Waterlogging in Tomato at e[CO(_2_)] by Multivariate Analysis. Int. J. Mol. Sci..

[17] Perreca E., Eberl F., Santoro M.V., Wright L.P., Schmidt A., Gershenzon J. (2022). Effect of Drought and Methyl Jasmonate Treatment on Primary and Secondary Isoprenoid Metabolites Derived from the MEP Pathway in the White Spruce *Picea glauca*. Int. J. Mol. Sci..

[18] Pietrak A., Salachna P., Lopusiewicz L. (2022). Changes in Growth, Ionic Status, Metabolites Content and Antioxidant Activity of Two Ferns Exposed to Shade, Full Sunlight, and Salinity. Int. J. Mol. Sci..

[19] Zhang T., Hong Y., Zhang X., Yuan X., Chen S. (2022). Relationship between Key Environmental Factors and the Architecture of Fruit Shape and Size in Near-Isogenic Lines of Cucumber (*Cucumis sativus* L.). Int. J. Mol. Sci..

[20] Yang C., Shi G., Li Y., Luo M., Wang H., Wang J., Yuan L., Wang Y., Li Y. (2022). Genome-Wide Identification of SnRK1 Catalytic alpha Subunit and FLZ Proteins in *Glycyrrhiza inflata* Bat. Highlights Their Potential Roles in Licorice Growth and Abiotic Stress Responses. Int. J. Mol. Sci..

[21] Du X., Lu Y., Sun H., Duan W., Hu Y., Yan Y. (2022). Genome-Wide Analysis of Wheat GATA Transcription Factor Genes Reveals Their Molecular Evolutionary Characteristics and Involvement in Salt and Drought Tolerance. Int. J. Mol. Sci..

[22] Wang S., Wang Y., Yang R., Cai W., Liu Y., Zhou D., Meng L., Wang P., Huang B. (2023). Genome-Wide Identification and Analysis Uncovers the Potential Role of JAZ and MYC Families in Potato under Abiotic Stress. Int. J. Mol. Sci..

[23] An B., Cai H., Li B., Zhang S., He Y., Wang R., Jiao C., Guo Y., Xu L., Xu Y. (2023). Molecular Evolution of Histone Methylation Modification Families in the Plant Kingdom and Their Genome-Wide Analysis in Barley. Int. J. Mol. Sci..

[24] Chen L., Cai M., Chen M., Ke W., Pan Y., Huang J., Zhang J., Peng C. (2022). Genome-Wide Characterization of PIN Auxin Efflux Carrier Gene Family in *Mikania micrantha*. Int. J. Mol. Sci..

[25] Li H., Chen L., Chen H., Xue R., Wang Y., Song J. (2022). The Role of Plant Progesterone in Regulating Growth, Development, and Biotic/Abiotic Stress Responses. Int. J. Mol. Sci..

[26] Liu H., Zeng B., Zhao J., Yan S., Wan J., Cao Z. (2023). Genetic Research Progress: Heat Tolerance in Rice. Int. J. Mol. Sci..

[27] Conti V., Parrotta L., Romi M., Del Duca S., Cai G. (2023). Tomato Biodiversity and Drought Tolerance: A Multilevel Review. Int. J. Mol. Sci..

